# Chemokine C–C motif ligand 2 overexpression drives tissue-specific metabolic responses in the liver and muscle of mice

**DOI:** 10.1038/s41598-020-68769-7

**Published:** 2020-07-20

**Authors:** Fedra Luciano-Mateo, Noemí Cabré, Salvador Fernández-Arroyo, Gerard Baiges-Gaya, Anna Hernández-Aguilera, Elisabet Rodríguez-Tomàs, Cristina Muñoz-Pinedo, Javier A. Menéndez, Jordi Camps, Jorge Joven

**Affiliations:** 1 Department of Medicine and Surgery, Universitat Rovira I Virgili, Reus, Spain; 2 Unitat de Recerca Biomèdica, Hospital Universitari Sant Joan, Institut D’Investigació Sanitària Pere Virgili, Reus, Spain; 3 Cell Death and Metabolism, Institut D’Investigació Biomèdica de Bellvitge, Barcelona, Spain; 4 Program Against Cancer Therapeutic Resistance (ProCURE), Metabolism and Cancer Group, Catalan Institute of Oncology, Girona, Spain; 5 Girona Biomedical Research Institute (IDIBGI), Girona, Spain; 6 The Campus of International Excellence Southern Catalonia, Tarragona, Spain

**Keywords:** Biochemistry, Cell biology, Diseases, Medical research, Molecular medicine

## Abstract

Chemokine (C–C motif) ligand 2 (CCL2) has been associated with chronic metabolic diseases. We aimed to investigate whether *Ccl2* gene overexpression is involved in the regulation of signaling pathways in metabolic organs. Biochemical and histological analyses were used to explore tissue damage in cisgenic mice that overexpressed the *Ccl2* gene. Metabolites from energy and one-carbon metabolism in liver and muscle extracts were measured by targeted metabolomics. Western blot analysis was used to explore the AMP-activated protein kinase (AMPK) and mammalian target of rapamycin pathways. *Ccl2* overexpression resulted in steatosis, decreased AMPK activity and altered mitochondrial dynamics in the liver. These changes were associated with decreased oxidative phosphorylation and alterations in the citric acid cycle and transmethylation. In contrast, AMPK activity and its downstream mediators were increased in muscle, where we observed an increase in oxidative phosphorylation and increased concentrations of different metabolites associated with ATP synthesis. In conclusion, *Ccl2* overexpression induces distinct metabolic alterations in the liver and muscle that affect mitochondrial dynamics and the regulation of energy sensors involved in cell homeostasis. These data suggest that CCL2 may be a therapeutic target in metabolic diseases.

## Introduction

Research on the factors that relate the immune system to metabolic alterations in chronic diseases is clinically important because it can allow the identification of therapeutic targets. It remains unclear how immunity affects systemic metabolism, but experimental evidence supports an intertwined relationship through interorgan metabolic crosstalk and mitochondrial dynamics^1–3^. One of the consequences of these processes is metabolic stress leading to adaptive responses and altered cellular communication^4^. Autophagy, a lysosomal degradation pathway, appears to be the most important effector in the adaptation to metabolic stress and removal of damaged organelles. Among the molecular sensors that modulate autophagy, the AMP-activated protein kinase (AMPK) and mammalian target of rapamycin (mTOR)-driven pathways play a crucial role in mitochondrial dysfunction and favor a bidirectional relationship between metabolism and inflammation^4,5^.

Chemokines, especially chemokine (C–C motif) ligand 2 (CCL2), have various functions that are involved in the maintenance of normal metabolism. CCL2 participates, directly and/or through the induced metabolic alterations, in the regulation of mitochondrial biogenesis and autophagy^6,7^. This chemokine also affects immune and inflammatory reactions and may compromise cell homeostasis and energy requirements in metabolic organs^8,9^. Growing evidence indicates that in both humans and experimental animals, the *Ccl2* gene is overexpressed in noncommunicable diseases characterized by a low degree of systemic inflammation and various metabolic alterations^10–14^. Recently, the relationships among mitochondrial dysfunction, autophagy and chronic diseases have been linked to CCL2^15^. Therefore, we hypothesized that CCL2 overexpression may actively contribute to the energy-related adaptive responses of metabolic organs through the regulation of intracellular sensors and signaling molecules. We then assessed the putative role of *Ccl2* in energy metabolism and one-carbon metabolism in cisgenic mice that systemically overexpress *Ccl2 (CgCcl2)*. Our results showed that the effects of *Ccl2* overexpression diverge in muscle and liver metabolism by inducing opposite alterations in energy and one-carbon metabolism, mitochondrial function and autophagy-related pathways.

## Results

### Variations in metabolic phenotype were associated with *Ccl2* overexpression

**Figure 1:**
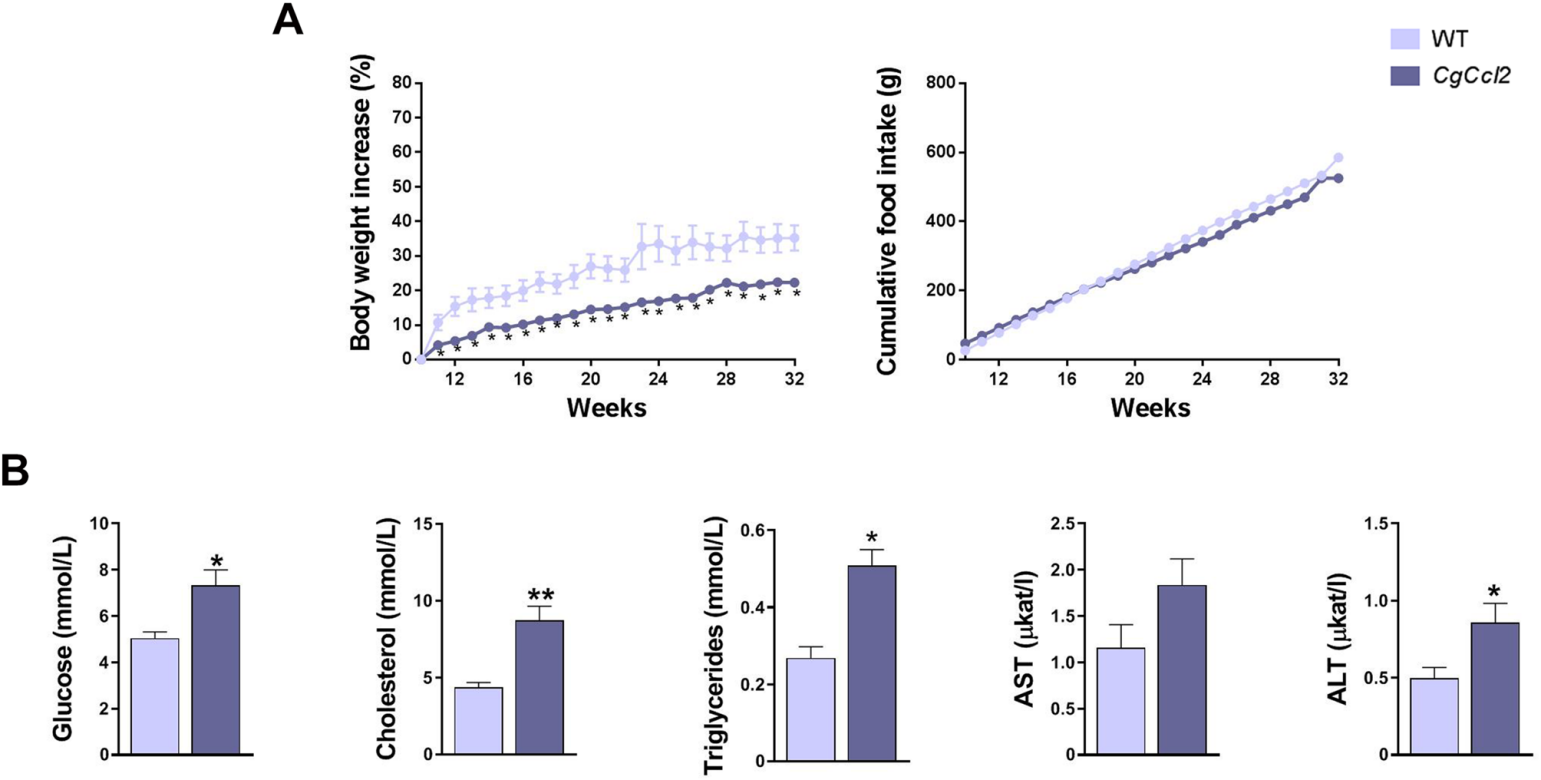
Selected metabolic features in the wild-type (WT) and *Ccl2* cisgenic mice (*CgCcl2*). The results are shown for (**A**) body weight increase and (**B**) biochemical variables and are shown as the mean ± SEM (n = 8 for each group). *P < 0.05; **P < 0.001, with respect to the WT control littermates.

**Figure 2:**
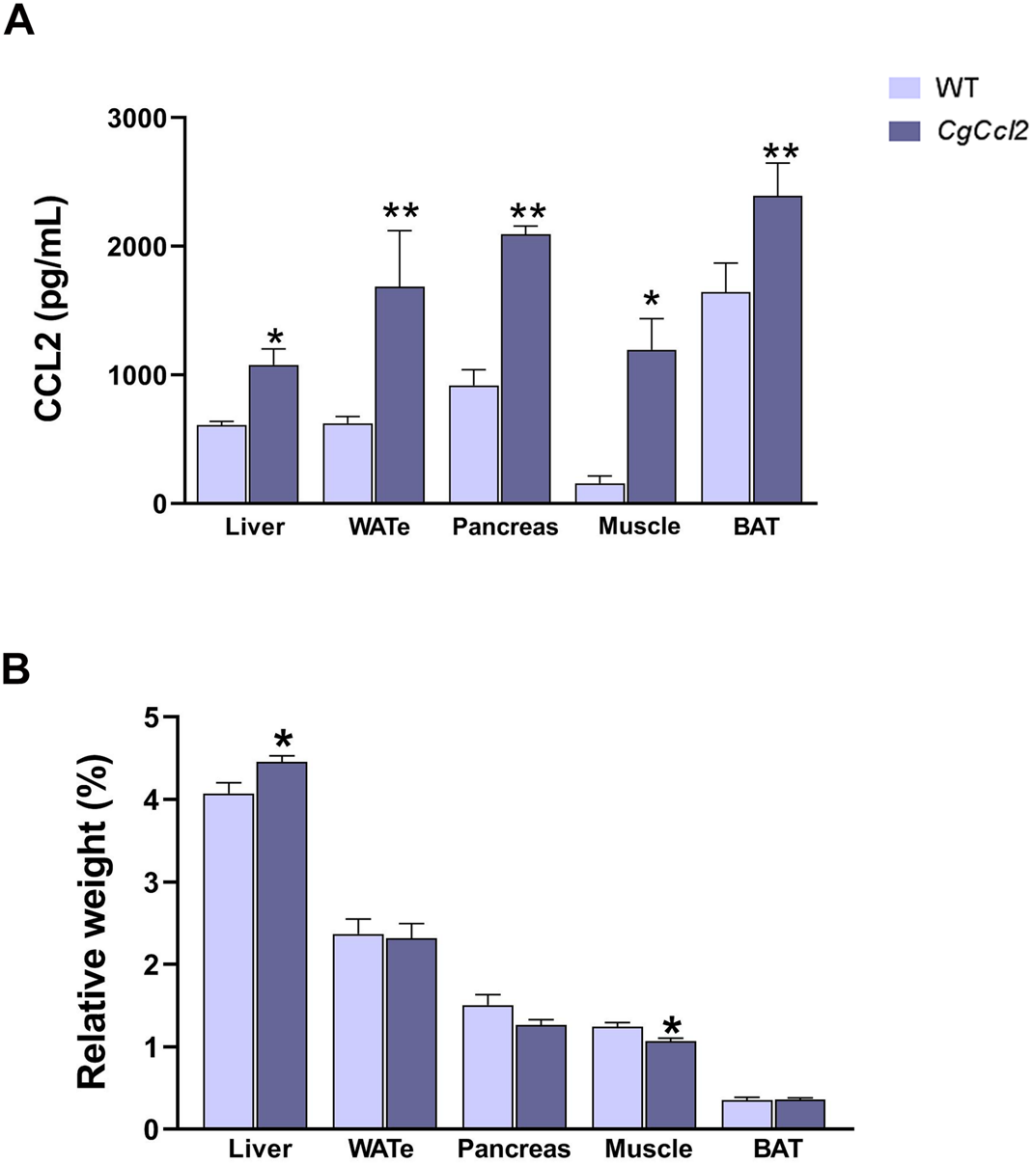
Increased *Ccl2* levels in the metabolic tissues from the *Ccl2* cisgenic mice (*CgCcl2*). The results are shown for (**A**) the *Ccl2* concentrations in the liver, white adipose tissue (eWAT), pancreas, muscle and brown adipose tissue (BAT) of the wild-type (WT) and *CgCcl2* mice and (**B**) the relative weight of the selected tissues. The results are shown as the mean ± SEM (n = 8 for each group). *P < 0.05; **P < 0.001, with respect to the WT control littermates.

**Figure 3:**
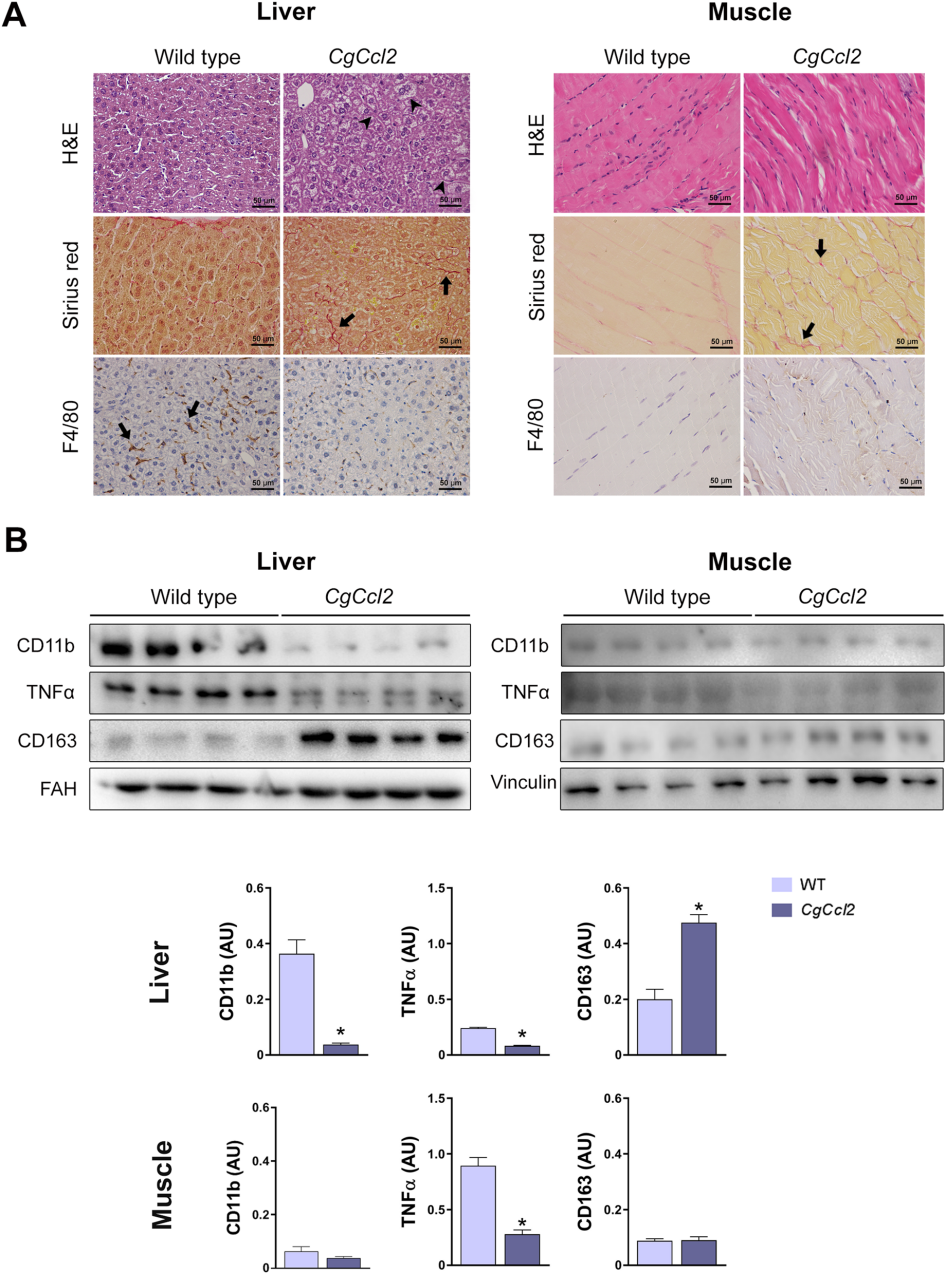
C*CL*2 overexpression induces histological alterations in the liver and muscle. (**A**) Representative microphotographs of the liver and muscle sections stained with hematoxylin and eosin (H&E), Sirius red and F4/80 marker. (**B**) Western blot analyses of clusters of differentiation (CD) 11b, tumor necrosis factor (TNFα), and CD163. Arrowheads indicate hepatic steatosis, and arrows show positive staining for Sirius red (collagen fibers) and F4/80. The results are shown as the mean ± SEM (n = 8). *P < 0.05, with respect to the WT control littermates.

All animals were matched for age. The *CgCcl2* mice did not show any significant differences in terms of frailty, behavior, reproduction or food intake compared to the wild-type (WT) mice. The body weight of these mice was lower than that of the WT mice, and there were significant alterations in the concentrations of serum glucose, cholesterol and triglycerides. The serum aspartate aminotransferase (AST) and alanine aminotransferase (ALT) activities of the *CgCcl2* mice were higher than those of WT mice (Fig. 1). As expected, the *CgCcl2* mice had higher CCL2 concentrations than the WT mice in all tissues examined, and *Ccl2* overexpression was associated with higher relative liver weight and lower relative muscle weight (Fig. 2). Histological analysis revealed that the *CgCcl2* mice accumulated lipid droplets in the liver and had muscle atrophy without major differences in other tissues. The liver and muscle tissues did not have any evidence of fibrosis and had fewer F4/80-stained cells (a marker of macrophages) than those of the WT animals (Fig. 3A). In the liver, the *CgCcl2* mice had lower expression of proinflammatory markers such as cluster of differentiation (CD) 11b and tumor necrosis factor-alpha (TNFα) and higher expression of CD163, which is an anti-inflammatory marker, than the WT mice. In muscular tissue, we found a significant decrease only in TNFα expression, but the levels of CD11b and CD163 in the *CgCcl2* mice were similar to those found in WT animals (Fig. 3B). Analysis of the expression of these genes by quantitative real-time polymerase chain reaction (qPCR) gave similar results (Supplementary Fig. S1).

### *Ccl2* overexpression induced opposite changes in mitochondrial quality and function in the liver and muscle

**Figure 4:**
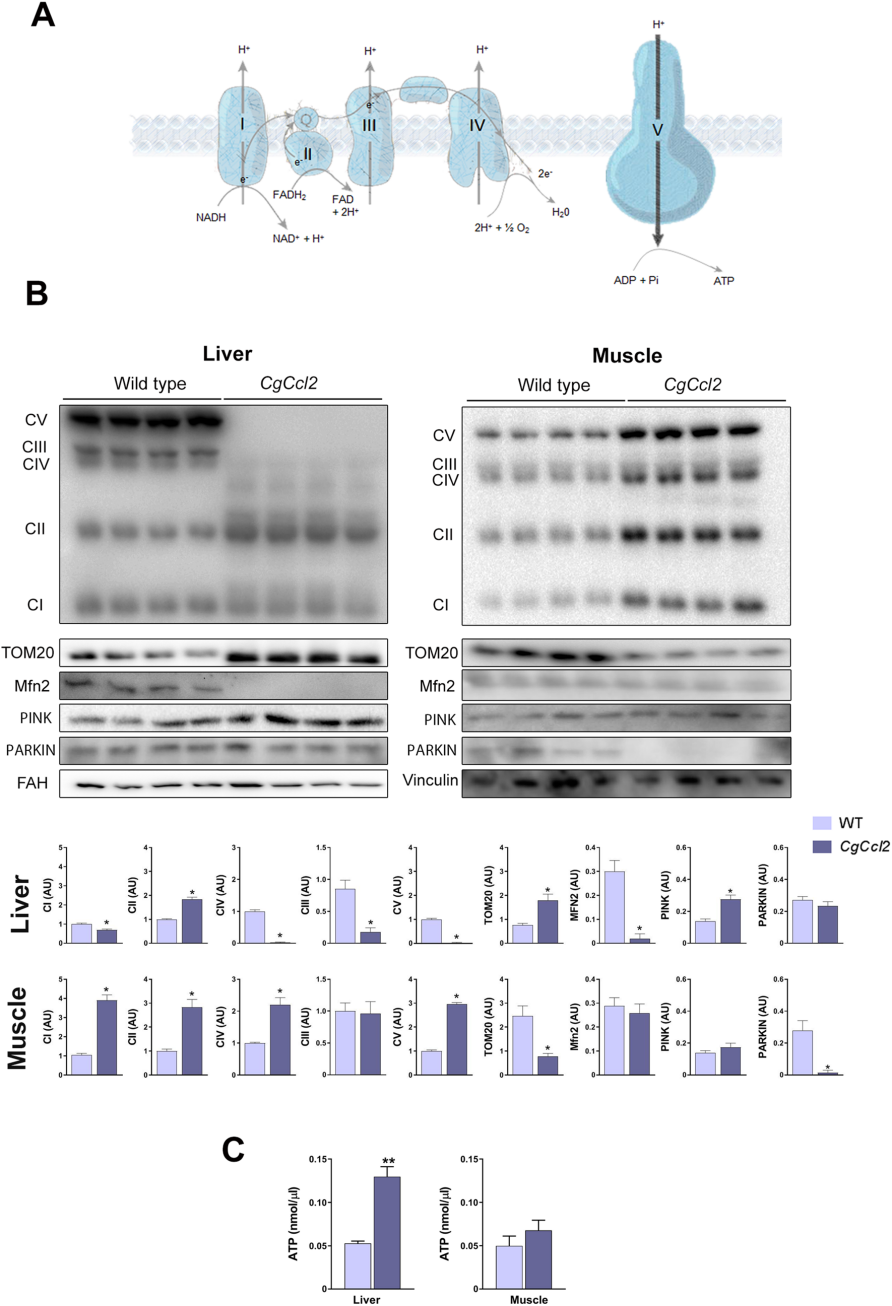
CCL2 affects the mitochondrial function. (**A**) Schematic representation of the oxidative phosphorylation complexes (OXPHOS). (**B**) Effects of CCL2 overexpression on OXPHOS, translocase of the outer membrane (TOM20), mitofusin 2 (MFN2), PTEN-induced kinase 1 (PINK1) and PARKIN. (**C**) ATP concentrations. The results are shown as the mean ± SEM. *P < 0.05 with respect to the WT control littermates.

To investigate whether *Ccl2* overexpression was associated with mitochondrial alterations, we assessed the expression of mitochondrial electron transport chain (ETC) complex proteins, as well as the mitochondrial import receptor subunit translocase of outer membrane 20 (TOM20), which is responsible for the recognition and translocation of cytosolically synthesized mitochondrial preproteins; mitofusin 2 (MFN2), which is required for mitochondrial fusion; and PTEN-induced putative kinase 1 (PINK1)/E3 ubiquitin- protein ligase parkin (PARKIN), two molecules involved in degradation of depolarized mitochondria. The *CgCcl2* mice had decreased levels of the oxidative phosphorylation complexes I (CI-NDUFB8), III (CIII-UQCRC2), IV (MTCO1), and V (CVNDUFB8) but not complex II (CII-SDHB); downregulation of MFN2 and upregulation of PINK1 without changes in PARKIN in the liver (Fig. 4A,B). PINK1 contributes to the identification of depolarized mitochondria, and PARKIN induces the degradation of damaged mitochondria by autophagolysosomes. In addition, the *CgCcl2* mice had an increase in hepatic mitochondrial density (a reflection of the number of mitochondria), which is consistent with the increase in TOM20 expression. The mitochondrial matrix was consistently less electrodense and the mitochondria were less interconnected and smaller than those in the WT mice (Supplementary Fig. S2). Therefore, our data suggest that the *CgCcl2* mice had a high number of mitochondria in the liver due to an inhibition of mitochondrial degradation. The hepatic concentration of ATP was increased (Fig. 4C). Conversely, *Ccl2* overexpression in the muscle resulted in enhanced levels of complexes I, II, IV, and V of the ETC and a decrease in TOM20 expression without any significant alterations in MFN2, PINK1 and ATP (Fig. 4).

### *Ccl2* overexpression induced different alterations in metabolites from energy and one-carbon metabolism in the liver and muscle

**Figure 5:**
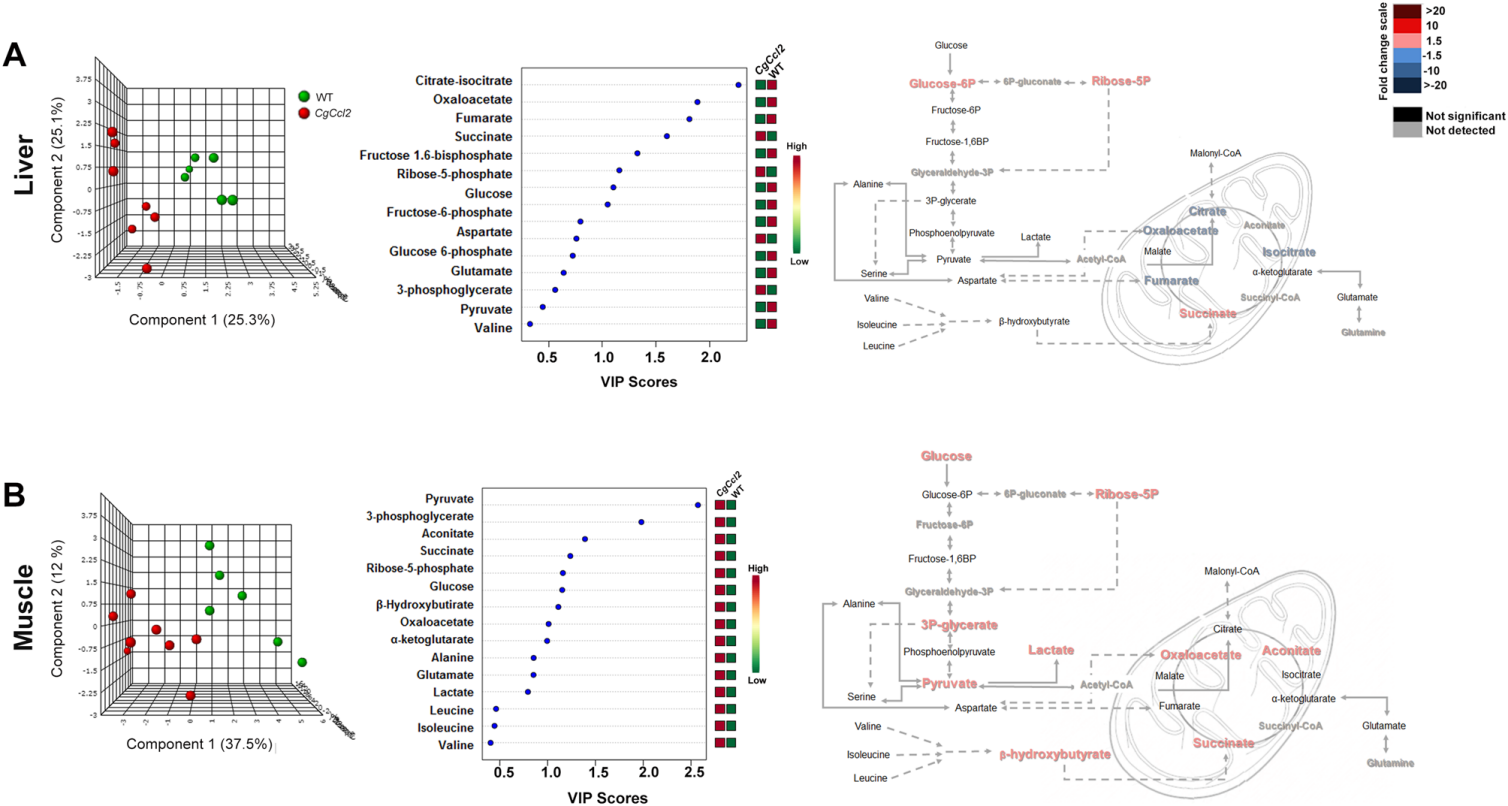
Effects of CCL2 overexpression on the levels of metabolites associated with energy metabolism. The results are shown for the liver (**A**) and muscle extracts (**B**) and include the partial least square discriminant analysis (PLS-DA), as well as random forest analysis and quantitative comparisons between the *Ccl2* cisgenic mice and the control littermates.

To explore pathways of energy adaptation in the mice with *Ccl2* overexpression compared to the controls, we used a targeted metabolomic approach to examine the metabolites from energy and one-carbon metabolism in the liver and muscle extracts (Supplementary Tables S1 and S2). There were differences between tissues and strains. In the liver, oxidative metabolism was relatively reduced in the *CgCcl2* mice with lower concentrations of citrate, oxaloacetate and fumarate, isolated accumulation of glucose-6-phosphate and probable alterations in the monophosphate shunt, likely indicating low replenishment of the citric acid cycle (CAC). Interestingly, liver succinate was increased without changes in amino acid metabolism, which suggests alterations in mitochondrial function. Partial least squares discriminant analysis (PLS-DA) and random forest analyses revealed major differences between the strains and showed that metabolites from CAC distinguished the mice with and without *Ccl2* overexpression (Fig. 5A). In contrast, in the muscle extracts, pyruvate provided the highest variable of importance score, denoting the relative accumulation of glucose, 3-phosphoglycerate, pyruvate, lactate, ribose 5-phosphate and β-hydroxybutyrate. Thus, anaplerosis-associated pathways were increased in the muscles of the *CgCcl2* mice compared to the WT mice, which correlated with the relative accumulation of metabolites from CAC, especially succinate, aconitate and oxaloacetate (Fig. 5B).

**Figure 6:**
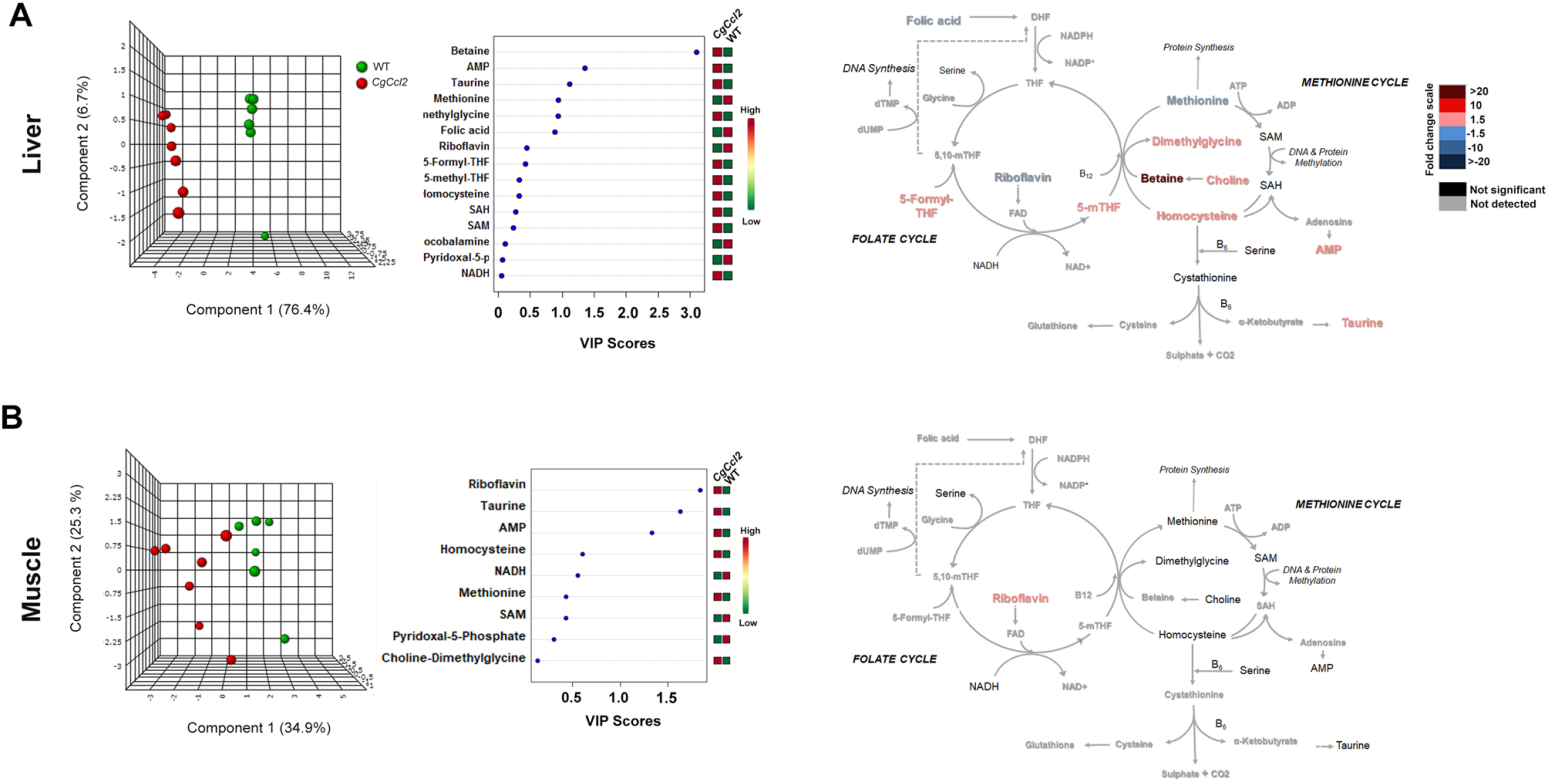
Effects of CCL2 overexpression on the levels of metabolites associated with one-carbon metabolism. The results are shown for the liver (**A**) and muscle extracts (**B**), and discrimination between strains was assessed via partial least square discriminant and random forest analyses.

PLS-DA and random forest analyses also revealed that metabolites from one-carbon metabolism segregated the strains according to *Ccl2* overexpression and highlighted major differences in the tissue-specific metabolic adaptations (Fig. 6). In the liver, alterations induced by *Ccl2* overexpression were consistent and mostly related to homocysteine transmethylation. Specifically, the depletion of methionine and the relative accumulation of choline, dimethylglycine, betaine, and 5-methyltetrahydrofolate strongly suggested an acquired dysfunction in betaine homocysteine methyltransferase (Fig. 6A). In contrast, one-carbon metabolism was not significantly altered in the muscle tissue (Fig. 6B).

### Ccl2 overexpression induced tissue-specific responses in autophagy-related pathways

**Figure 7:**
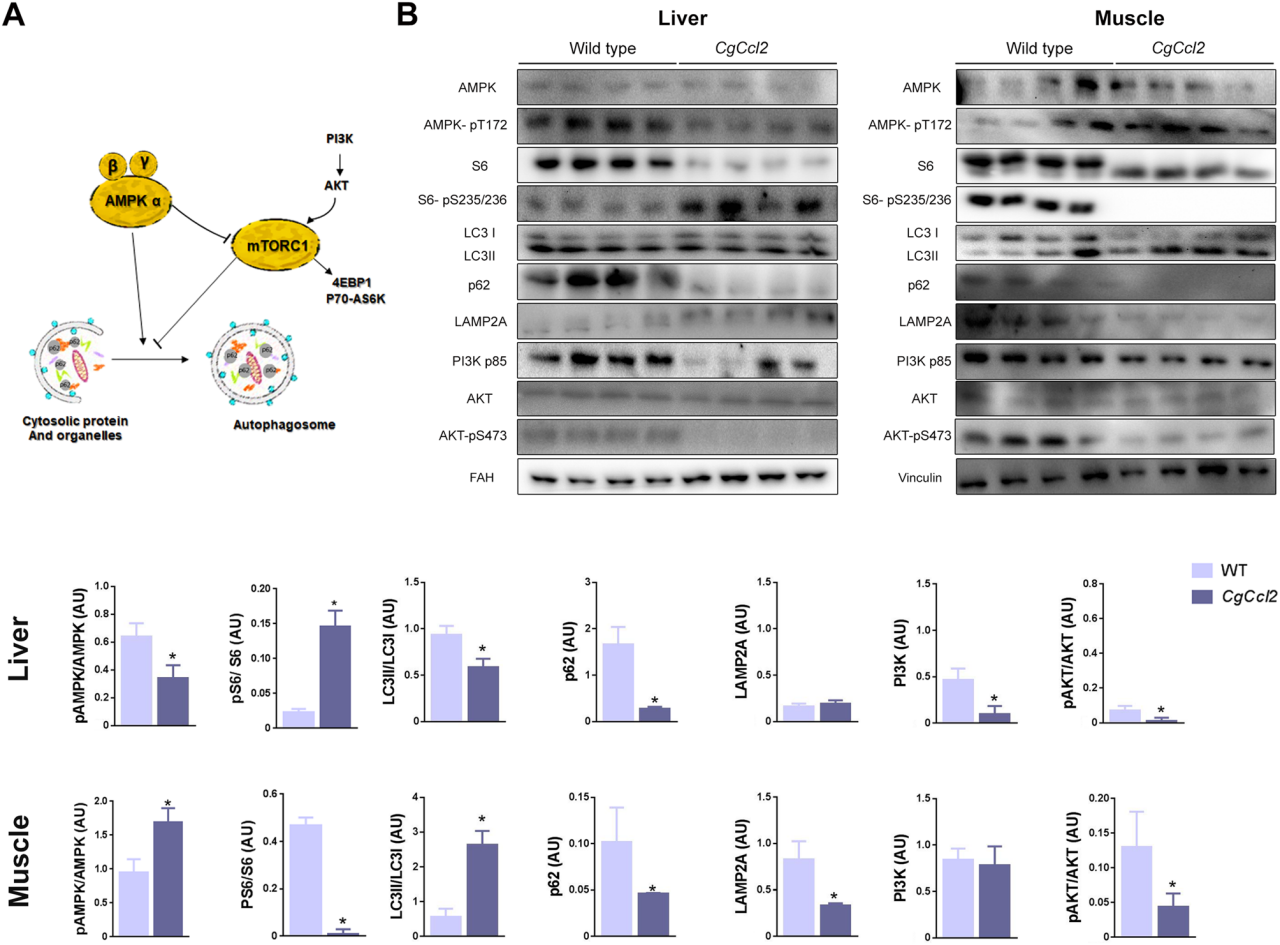
Effects of CCL2 on autophagy-related pathways. (**A**) Schematic overview and (**B**) overall assessment of expression levels measured by Western blot analysis of selected molecules. The results are shown as the mean ± SEM. *P < 0.05, with respect to the WT control littermates. *PI3K-p85* phosphoinositide 3-kinase p85 subunit, *AKT* protein kinase B, *S6* ribosomal protein, *S6-Ps235/236*, phospho-S6 ribosomal protein, *AMPK* AMP-activated protein kinase, *LC3* microtubule-associated protein 1A/1B light chain 3B, *p62/SQSTM1* sequestosome 1, *LAMP2A* lysosome-associated membrane protein 2, *FAH* fumarylacetoacetate hydrolase.

Autophagy participates in the elimination of damaged mitochondria, has strong anti-inflammatory effects and improves energy homeostasis. Increased CCL2 levels were shown to influence autophagy-mediated cell fate decisions activating mTORC1 through simultaneous regulation of both the AMPK and protein kinase B (AKT) pathways^15^. Therefore, we explored the potential impact of *Ccl2* overexpression in the liver and muscle on these metabolic sensors. In the liver, *Ccl2* overexpression decreased AMPK activity and upregulated mTORC1 compared to those in the WT mice, as indicated by the lower AMPK phosphorylation and higher ribosomal protein S6 phosphorylation, respectively. The decrease in the ratio between forms II and I of the microtubule-associated protein 1A/1B light chain 3B (LC3II/LC3I ratio) and the reduced expression of sequestosome 1 (p62/SQSTM1) without changes in the lysosome-associated membrane protein 2 (LAMP2A) levels strongly indicated decreased autophagy in the livers of the *CgCcl2* mice. The reduction in autophagosome formation was confirmed by alterations in phosphoinositide 3-kinase (PI3K-p85) and AKT-pS473, which indicated dysfunction in the insulin growth factor pathway (Fig. 7A,B). In muscle from the *CgCcl2* mice, autophagy was activated compared to that in the WT mice, in which AMPK phosphorylation and the LC3II/LC3I ratio were increased, although the p62/SQSTM1 levels were decreased. Notably, LAMP2A expression in the *CgCcl2* mice was significantly decreased, indicating a potential effect on chaperone-mediated autophagy without any effect on PI3K-p85 expression or the pAKT/AKT ratio (Fig. 7B).

## Discussion

Recent data have demonstrated that the biological function of *Ccl2* extends far beyond the recruitment of monocytes and the overall inflammatory response. *Ccl2* protein and mRNA expression may be found in most cells and virtually all tissues^16,17^, and this wide distribution suggests a coordinated role in adaptive responses to systemic injuries. *Ccl2* signals through G protein-coupled receptors may strongly affect metabolism and have been accepted as a potential link between immune and metabolic functions underlying human chronic diseases^18–23^. High circulating levels of CCL2 correlate with biological age in mammals and are closely associated with a deleterious course of chronic diseases^24–27^. Our findings indicated that CCL2 may be locally involved in multiple metabolic pathways and, more importantly, shows paracrine effects with different outcomes in important metabolic organs^28^. Indeed, our study showed that overexpression of CCL2 in mice is associated with increased liver and decreased muscle weights, which largely mimics a phenotype frequently found in some human metabolic diseases, such as obesity, chronic liver disease or metabolic syndrome, and in aging. Metabolically active CCL2 is secreted by senescent cells^29^, and *Ccl2* overexpression can have positive or negative effects, depending on the context. In our model, the decreased expression of the F4/80 antigen in liver and muscle induced a shift towards decreased pro-inflammatory activity. A previous study by our group already showed that the *CgCcl2* mice had decreased expression of the F4/80 antigen and the chemokine receptor CCR7, inducing a shift towards lower pro-inflammatory activity^13^. The reasons for these findings are not known, but other authors reported a dual dose-dependent effect (pro- or anti-inflammatory) of CCL2 both in vitro and in vivo*.* These researchers suggested that low levels of CCL2 could be anti-inflammatory, while high levels would be pro-inflammatory. This dual effect of CCL2 would be independent of its effects on metabolism^30,31^. In mice, high *Ccl2* expression for a protracted period of time increases the expression of anti-inflammatory genes in metabolic tissues and may reduce the inflammatory response, but this effect may differ among organs^32^.

This anti-inflammatory response did not counteract the observed metabolic damage in liver and muscle caused by *Ccl2* overexpression, indicating the active role of CCL2 in cell homeostasis. Indeed, targeting CCL2 and other factors secreted by senescent cells alleviated metabolic dysfunction and showed efficacy in diabetes and obesity^33^. In the present study, we observed different effects of CCL2 in the liver and muscle of mice. In the liver, *Ccl2* overexpression resulted in steatosis, decreased AMPK activation, and upregulation of mTORC1. These alterations were associated with decreased oxidative phosphorylation and alterations in the citric acid cycle and transmethylation pathways. In contrast, in the muscle, we found increased AMPK activity and mTORC1 downregulation, together with an increase in oxidative phosphorylation processes and increased concentrations of β-hydroxybutyrate and lactate. Our data suggest a bidirectional interaction between CCL2 and mitochondrial function, which might explain the different changes in muscle and liver metabolism as a result of their distinct energy requirements. The effect of *Ccl2* overexpression may be linked to alterations in mitochondrial dynamics and the AMPK-driven pathways that regulate anabolic and catabolic pathways^34–36^. In particular, CCL2 appears to specifically affect the response in energy and one-carbon metabolism and autophagic processes with important repercussions on energy status. *CgCcl2* mice showed an anabolic profile in the liver, decoupling of oxidative phosphorylation components and alterations in mitochondrial fusion, which have been previously related to liver disease^37,38^. The response diverged in skeletal muscle, which had with a catabolic profile, increased expression of oxidative phosphorylation components and increased levels of lactate and ketone bodies without alterations in mitochondrial fusion markers. We found elevated levels of ATP in the livers of the *CgCcl2* mice despite mitochondrial alterations and OXPHOS inhibition. This finding could be explained by the low expression of MFN2. Previous studies^39^ reported that MFN2 loss-of-function causes a specific alteration in the expression of subunits that participate in complexes I, II, III and V, which leads to reduced activity of several components of the OXPHOS system. Under these conditions, MFN2 inhibition induces a higher rate of glucose uptake and glycolysis and a lower rate of glycogen synthesis, thus compensating for cell energy metabolism.

Metabolomic analysis showed that the *CgCcl2* mice had different responses in the liver and muscle with respect to the accumulation of metabolites from one-carbon metabolism with likely metaboloepigenetic effects^40^. The differences were also evident in the response of metabolites from the CAC that may induce mitochondrial dysfunction through G-protein-coupled receptor signaling^41^. Some of these receptors may be blocked and have been assayed in the treatment of inflammatory conditions, but the associated metabolic events are contradictory and diverse among the involved tissues^42,43^. Our findings certainly illustrate the expected difficulties in the assessment of effects derived from the immune regulation of metabolic homeostasis and may partially explain the heterogeneous responses of AMPK stimulators among cells and organs^13,43^.

In conclusion, *Ccl2* gene overexpression can affect processes that are important for cell survival and homeostasis, including mitochondrial dynamics and autophagy-related pathways. The differential response in tissues with different energetic needs may complicate the assessment of therapeutic strategies designed to block CCL2 metabolic signaling.

## Materials and methods

### Animals and experimental design

We employed male *Ccl2* cisgenic mice with a C57BL/6J genetic background that systemically overexpress *Ccl2*. These animals have an additional copy of the *Ccl2* gene in the Gt(ROSA)26Sor locus in the mouse genome and were obtained via recombination in embryonic stem cells^13^. The strain was backcrossed for > 10 generations to maintain homozygosity, and littermates without mutations were used as controls (WT). All mice were maintained in a facility with a controlled temperature (22 °C), humidity (50%) and light/dark cycle (12/12 h). The mice were fed a normal standard diet from Scientific Animal Food and Engineering (SAFE, Augy, France) and water at libitum for 22 weeks. The animal procedures were designed according to European guidelines (Directive 2010/63/EU) and approved by the Ethics Review Committee for Animal Experimentation of *Universitat Rovira i Virgili* (protocols 4815 and GC-URV-0235-03.18.2014).

### Sample collection and laboratory measurements

At sacrifice, blood was obtained by intracardiac puncture to measure serum glucose, cholesterol, and triglyceride concentrations and AST and ALT activities with a Roche Cobas Mira Plus automated analyzer (Roche Diagnostics, Basel, Switzerland). Portions of liver, epididymal white adipose tissue (eWAT), interscapular brown adipose tissue (BAT), pancreas and soleus/gastrocnemius skeletal muscle were frozen in liquid N_2_ and stored at − 80 °C and/or fixed in formalin (3.7–4% formaldehyde buffered to pH 7 and stabilized with 1–1.5% methanol) until analysis. Thirty milligrams of the selected tissues was homogenized by using a sonicator (Branson Sonifer 150, Thistle Scientific, Glasgow, UK) in 50 mM Tris with 1 mM EDTA, 1% Igepal CA-630, 150 mM NaCl, 0.10% Triton, 50 mM NaF, 100 mM phenylmethanesulfonyl fluoride and 1 mM Na_3_VO_4_. To measure the CCL2 concentrations in liver, pancreas, muscle, and white and brown adipose tissues by ELISAs (Preprotech, London UK), we used two hundred micrograms of protein from each tissue.

### Histological analyses and immunohistochemistry

Two-micrometer-thick tissue sections were stained with hematoxylin and eosin and Sirius red to evaluate the histological alterations. For the immunohistochemical analyses, we used 10 mM Tris, pH 6, with 1 mM EDTA for antigen retrieval in a microwave oven at 90 °C for 30 min. Bovine serum albumin (2%) was used to block nonspecific binding. Endogenous peroxidases were blocked by hydrogen peroxide. Then, the sections were incubated with an antibody against F4/80 (ab100790, Abcam, Cambridge, UK), which is a marker of macrophages, and a secondary antibody (P0448, Dako, Agilent, CA, USA). All sections were counterstained with Mayer’s hematoxylin.

### Analysis of the morphology of mitochondria by transmission electron microscopy

Small pieces of the liver were immediately fixed in a 2% glutaraldehyde solution in 0.1 M cacodylate buffer, pH 7.4. The samples were then postfixed in 1% osmium tetroxide for 2 h and dehydrated in sequential steps of acetone prior to impregnation in increasing concentrations of the resin in acetone over a 24 h period. Semithin sections (500 nm) were stained with 1% toluidine blue. Ultrathin sections (70 nm) were subsequently cut using a diamond knife, double-stained with uranyl acetate and lead citrate, and examined using a transmission electron microscope (Hitachi, Tokyo, Japan). Mitochondrial density, elongation and interconnectivity were quantified using the method of Dagda et al.^44^.

### Western blot analyses

Twenty milligrams of frozen liver and skeletal muscle tissues from each animal was homogenized by using a sonicator (Branson Sonifer 150). For hepatic tissue, we used 300 μL of lysis buffer composed of 0.25 M sucrose, 1 mM Pefabloc SC (Sigma-Aldrich, Saint Louis, MI, USA), and a phosphatase inhibitor cocktail (Hoffman-La Roche, Basel, Switzerland). For muscle tissue, we used 300 μL of lysis buffer composed of 50 mM Tris, 1 mM EDTA, 1% Igepal CA-630, 150 mM NaCl, 0.1% Triton, 50 mM NaF, 100 mM phenylmethanesulfonyl fluoride and 1 mM Na_3_VO_4_. Western blotting was performed by denaturing 50 μg of protein at 100 °C for 5 min in Laemmli sample buffer and β-mercaptoethanol. For protein separation, an 8–14% sodium dodecyl sulfate–polyacrylamide gel was used, and the proteins were transferred onto a polyvinylidene difluoride or nitrocellulose membrane (Thermo Fisher, Barcelona, Spain). Before the primary antibody incubation, the membranes were blocked with 5% nonfat milk or bovine serum albumin in Tris, sodium chloride and 1% Tween-20 (pH = 7.4). Other information on the reagents is shown in Supplementary Table S3. The bands were detected with SuperSignal West Femto chemiluminescent substrate (Pierce, Rockford, IL, USA), and the analysis was performed with a ChemiDoc system (Bio-Rad Laboratories, Madrid, Spain). The bands were analyzed and quantified using Image Lab 2.0 software (Bio-Rad Laboratories, Hercules, CA, USA). We explored the expression of proinflammatory and anti-inflammatory markers and molecules involved in the regulation of energy metabolism, autophagy and lysosomal function^14,45–48^. Measurements were performed for CD11b, CD163, TNFα, phosphoinositide 3-kinase p85 subunit (PI3K-p85), AKT, phospho-AKT Ser 473 (AKT-pS473), S6, phospho-S6 ribosomal protein (S6-Ps235/236), TOM20, MFN2, AMPK, AMPK-pT172, PINK1, PARKIN, the oxidative phosphorylation complexes LC3I and LC3II, p62/SQSTM1 and LAMP2A. Fumarylacetoacetate hydrolase (FAH) and vinculin were used as reference proteins. Details on the source and handling of the antibodies are described in Supplementary Table S3. The uncropped blots are shown in Supplementary Fig. S3.

### ATP assay

ATP concentrations were analyzed by an ATP assay kit (ab83355, Abcam, Cambridge, UK) according to the manufacturer’s instructions. Briefly, 60 mg of liver and skeletal muscle was homogenized using a Dounce homogenizer in ATP assay buffer (Izasa, Barcelona, Spain). The homogenates were centrifuged at 13,000*g* for 5 min at 4 °C and deproteinized with trichloroacetic acid (Deproteinizing Sample Preparation kit, ab204708, Cambridge, UK).

### Quantitative real-time polymerase chain reaction

Total RNA was extracted using an RNeasy kit (Qiagen, Barcelona, Spain) and was reversed transcribed using a Reverse Transcription System kit (Applied Biosystems; Invitrogen, Barcelona, Spain). Quantitative real-time PCR (qPCR) was conducted on a 7900HT Fast Real-Time PCR System using TaqMan Gene Expression Assays (Applied Biosystems). The results were normalized according to the expression level of Beta-2-Microglobulin (B2M) mRNA.

### Measurement of energy balance and one-carbon metabolites in the liver and muscle tissue

The settings and conditions used for the instrumentation in the metabolomics analyses have been previously described^49,50^. Briefly, for quantification of the metabolites involved in energy generation, 10 mg of liver and skeletal muscle was homogenized using a Precellys 24 homogenizer (Izasa, Barcelona, Spain) in 0.5 mL of methanol (MeOH)/H_2_O (8:2 *v/v*) containing D_4_-succinic acid as an internal standard at a final concentration of 0.01 μM. The samples were stored at − 20 °C for 2 h to precipitate the proteins and then centrifuged at 15,000 rpm for 10 min at 4 °C. The supernatants were collected, dried under N_2_ flow and derivatized with methoxyamine hydrochloride dissolved in pyridine (40 mg/ml) and N-methyl-N-trimethylsilyl trifluoroacetamide. The analyses were performed with a 7890A gas chromatograph coupled via an electron impact source to a 7,200 quadrupole time-of-flight mass spectrometer (GC-EI-QTOF-MS) equipped with a J&W Scientific HP-5MS column (30 m × 0.25 mm, 0.25 μm) (Agilent Technologies, Santa Clara, CA, USA).

For quantification of the intermediaries involved in one-carbon metabolism, 0.5 mL of MeOH:H_2_O (8:2 *v/v*) containing 1% ascorbic acid (*m/v*) and 0.5% β-mercaptoethanol (*v/v*) was added to 10 mg of liver and skeletal muscle and homogenized. After protein precipitation for 2 h at − 20 °C, the samples were centrifuged at 15,000 rpm at 4 °C for 10 min, the supernatant was collected, and the homogenization step was repeated. Nonpolar metabolites were removed by adding 2 mL of chloroform/methanol (2:1, *v/v*) to prevent interference in the analysis. After 10 min of agitation, the samples were centrifuged at 15,000 rpm for 10 min at 4 °C, and the polar phase was collected, dried under N_2_ flow and stored at − 80 °C until analysis. The samples were then resuspended in 100 µL of ultrapure type 1 water containing 50 mM ammonium acetate plus 0.2% formic acid and placed into chromatographic vials for analysis by a 1,290 Infinity ultra-high-performance liquid chromatography instrument coupled to a 6,490 triple quadrupole-mass spectrometer using an electrospray ionization source (Agilent Technologies) and equipped with an Acquity UPLC HSS T3 column (2.1 × 150 mm, 1.8 μm) (Waters Corporation, Milford, MA, USA).

### Statistical analyses

The statistical analyses were performed using nonparametric Mann–Whitney *U*-tests with IBM SPSS Statistics for Windows, Version 21 (Armonk, NY, USA, IBM Corp.). Selected metabolites were quantitated across all the samples using MassHunter Quantitative Analysis B.07.00 (Agilent Technologies) according to the calibration curve of its corresponding standard. The PLS-DA analysis and the variable importance in projection (VIP) score were calculated with MetaboAnalyst 4.0 (https://www.metaboanalyst.ca) software^51^. Differences were considered statistically significant when the *P* value was ≤ 0.05. Unless otherwise indicated, the results are expressed as the mean ± standard error of the mean.

## Supplementary information

Supplementary file1 (PDF 2148 kb)
