# Supplementary material for: Chemokine C–C motif ligand 2 overexpression drives tissue-specific metabolic responses in the liver and muscle of mice
Source: Sci Rep. 2020 Jul 20;10:11954. doi: 10.1038/s41598-020-68769-7 (PMC7371894; doi:10.1038/s41598-020-68769-7)

Supplementary materials corresponding to:

Chemokine C-C motif ligand 2 overexpression drives tissue-specific metabolic responses in the liver and muscle of mice

Fedra Luciano-Mateo [1,2], Noemí Cabré [1,2], Salvador Fernández-Arroyo [1,2], Gerard Baiges-Gaya [2], Anna Hernández-Aguilera [1,2], Elisabet Rodríguez-Tomàs [2], Cristina Muñoz-Pinedo [3], Javier A. Menéndez [4,5], Jordi Camps [1,2], Jorge Joven [1,2,6]

[1] Universitat Rovira i Virgili, Department of Medicine and Surgery, Reus, Spain. [2] Unitat de Recerca Biomèdica, Hospital Universitari Sant Joan, Institut d'Investigació Sanitària Pere Virgili, Reus, Spain. [3] Cell Death and Metabolism, Institut d'Investigació Biomèdica de Bellvitge, Barcelona, Spain. 4 Program Against Cancer Therapeutic Resistance (ProCURE), Metabolism and Cancer Group , Catalan Institute of Oncology, Girona , Spain. [5] Girona Biomedical Research Institute (IDIBGI), Girona , Spain. [6] The Campus of International Excellence Southern Catalonia, Tarragona, Spain. Correspondence and requests for materials should be addressed to J.C. (email: jcamps@grupsagessa.com) or J.J. (email: jjoven@grupsagessa.com).

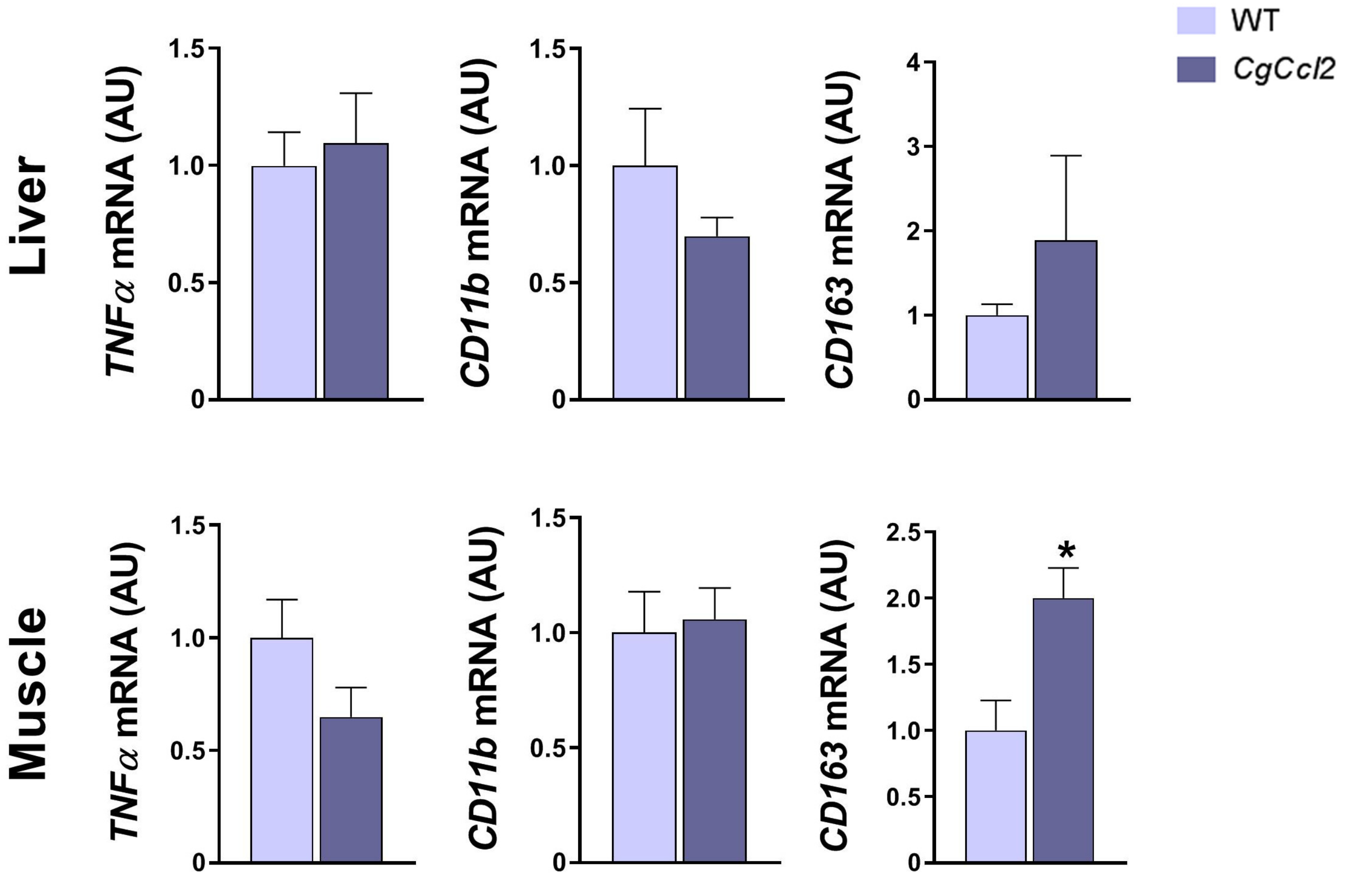

Supplementary Figure S1. Gene expression analyses of tumor necrosis factor-α (TNFα) and cluster of differentiation (CD) 11b and 163 in wild type (WT) and cisgenic mice (*CgCccl2*). *$P < 0.05$, with respect to WT mice.

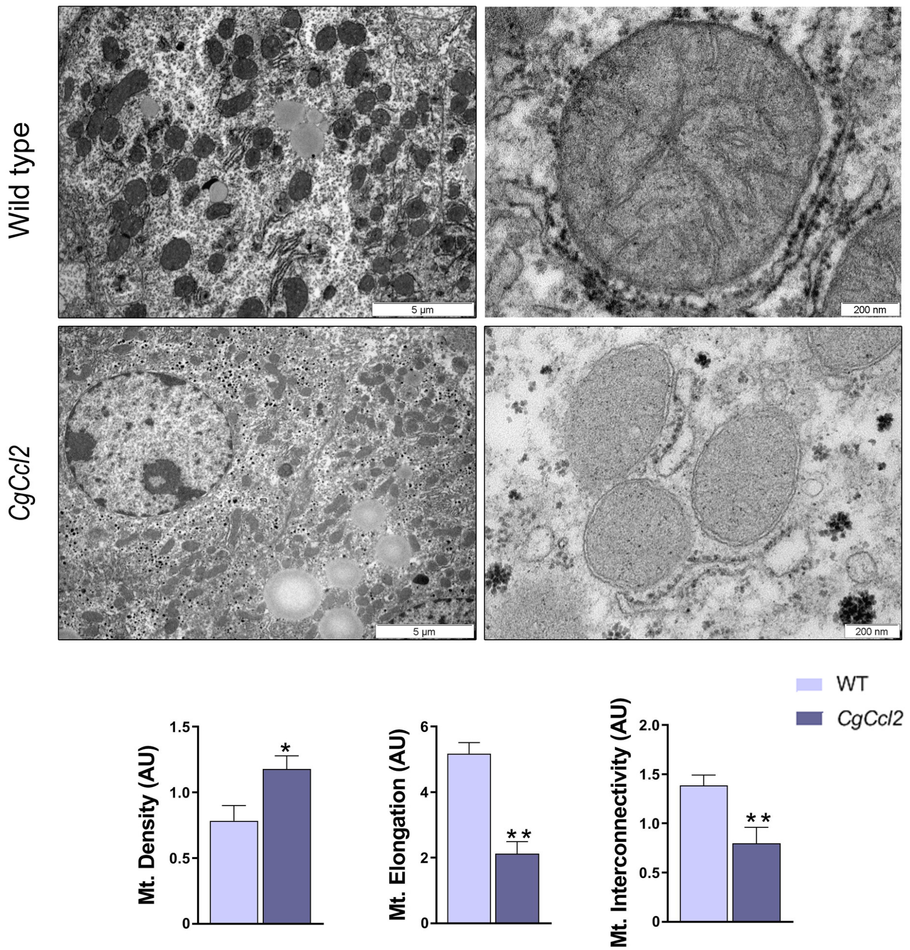

Supplementary Figure S2. Transmission electron microscopy analysis of mitochondria in the liver of wild type (WT) and cisgenic mice (*CgCcl2*). Mt.: Mitochondria. *$P < 0.05$; **$P < 0.01$, with respect to WT mice.

Supplementary Table S1. Hepatic and muscle energy-balance metabolites. Values are shown in μM/ 100 mg of tissue (mean and interquartile range). * $P < 0.05$, respect to wild type group. The lack of values denotes a significant number of observations under the limit of quantification.

	Liver		Muscle	
**Metabolites**	**Wild type**	***CgCcl2***	**Wild type**	***CgCcl2***
**Glucose**	53097.7 (42523.5-68695.5)	44395.8 (16996-55033.1)	373.4 (345-394.2)	726.3 (681.4-1314.1)*
**Glucose 6-phosphate**	45.1 (39.1-48.3)	103.6 (48.7-132.7)*	16 (12.8-18.8)	15.6 (10-25.8)
**Fructose-6-phosphate**	428.4 (258.9-552.7)	391.5 (136.6-474)	-	-
**Fructose 1.6-bisphosphate**	63.4 (52.3-85.7)	60.6 (11.8-94.4)	3.6 (2.4-13.4)	7 (2.9-10.8)
**3-phosphoglycerate**	15.7 (14.7-22.1)	22.3 (17.2-25)	1.9 (1.6-3.2)	5.8 (2.6-7.9)*
**Phosphoenolpyruvate**	8.1 (6.1-15.6)	9 (8.3-12.3)	0.5 (0.3-1.1)	0.5 (0.5-0.7)
**Pyruvate**	422 (325.7-570.5)	337.8 (247.5-491.5)	68.8 (54.8-221.2)	597.3 (499.3-675.2)*
**Lactate**	16199.3 (14922.1-16470.7)	14521.1 (12177.8-18810.5)	9815.7 (6626.5-16649.8)	17001 (8834.4-27368.7)*
**Ribose-5-phosphate**	27.6 (24.5-32.3)	55.1 (51.7-71)*	9 (7.8-10.7)	18.4 (16.6-30.2)*
**Oxaloacetate**	946.7 (701.9-1143.8)	464 (222.4-821.4)*	27.7 (15.7-33.4)	58.9 (48.4-66.9)*
**Citrate-isocitrate**	28.9 (14.9-39.8)	8.9 (6.8-17)*	51.6 (27.1-119.4)	53.1 (30.5-125.6)
**Aconitate**	-	-	11.5 (10.3-12.9)	35.8 (14.8-56.4)*
**α-ketoglutarate**	21.2 (15.2-26.9)	18.9 (10.7-28)	2.1 (1.2-5.1)	4.5 (1.2-7.8)
**Succinate**	382.9 (247.7-411.5)	471.5 (386.4-563.4)*	41.2 (38.1-79.1)	110.3 (110.3-126.8)*
**Fumarate**	485.3 (405.2-686.8)	323.4 (273.9-330.9)*	143.7 (56.9-217.6)	160 (116-167.3)
**Malate**	1191.6 (932.2-1552.4)	825.4 (614.7-1408)	322.2 (133.8-502)	373.3 (308.4-379.9)
**β-Hydroxybutirate**	216.4 (162.2-250)	215.8 (168.6-288.2)	38.4 (22-51.3)	76.1 (76.1-84.8)*
**Glutamate**	10659.9 (8844.7-15265.4)	9457.2 (6229.3-12508.3)	1673 (1212.6-2498.8)	2648.6 (1750.1-4824.8)
**Alanine**	515.8 (466.5-706.2)	517.3 (396.7-656.6)	362.2 (286.4-414.6)	508.2 (432.2-1155.3)
**Serine**	2282.2 (2261.4-2327.6)	2253.8 (2045.9-3281)	1007.8 (591.2-1375)	1008.1 (660.7-1749.1)
**Valine**	551.7 (469.8-702.5)	622.1 (339.3-788.6)	123.3 (75.9-201.2)	158.8 (95.9-252.4)
**Isoleucine**	568.6 (552.6-585.7)	603.8 (442.2-790.6)	77.1 (45-116.7)	101.3 (55.6-181)
**Leucine**	1440.7 (1398.9-1469.4)	1509.9 (1248.6-1886.2)	163.8 (102-260.6)	262.3 (124.5-389.6)
**Aspartate**	253.9 (226.8-332.6)	201.6 (154.9-276.5)	245 (108.4-374.3)	258.6 (207.4-343.2)

Supplementary Table S2. Hepatic and muscle one-carbon metabolites. Values are shown in µM/ 100 mg of tissue (mean (interquartile range), except (**‡**) where are expressed in nM/100 mg of tissue**.** * *P* < 0.05, ***P* < 0.001 respect to wild type group. The lack of values denotes a significant number of observations under the limit of quantification.

	Liver		Muscle	
**Metabolites**	**Wild type**	***CgCcl2***	**Wild type**	***CgCcl2***
**Cystathionine‡**	6.7 (6.2-11.2)	8.8 (6.9-8.9)	-	-
**Taurine**	494.2 (274.4-541.1)	1027.7 (884.6-1582.1)*	1499.7 (601.8-3621.9)	2385.2 (1811.5-5839.9)
**Choline-Dimethylglycine**	13.9 (8.1-15.5)	36.9 (36.6-47.6)*	0.1 (0.1-0.2)	0.1 (0.1-0.2)
**Betaine‡**	15 (13.2-15.2)	1012.5 (448.6-1497.7)**	-	-
**Homocysteine**	0.7 (0.6-0.8)	1.1 (1-2.4)*	0.3 (0.3-0.4)	0.4 (0.2-0.7)
**SAM**	1.2 (0.9-1.4)	1.5 (1.5-1.6)	3.3 (1.7-4.5)	2.7 (2.3-2.9)
**Methionine**	42.1 (41-42.7)	9.9 (8.7-19.2)**	0.6 (0.6-0.7)	0.7 (0.3-1.2)
**AMP**	57.4 (56.1-61.8)	338.8 (338.8-407.9)**	3.7 (2.1-5.7)	7.7 (3.8-10.7)
**NADH**	12.5 (10.9-14.6)	13.8 (13-14.4)	1.7 (1.3-2.5)	1.5 (0.7-2.5)
**SAH‡**	33.5 (24.7-46.2)	52.1 (29.2-75.1)	-	-
**Pyridoxal 5 phosphate**	12.5 (12.5-12.5)	10.3 (9.4-14.1)	3.5 (2.2-7.1)	3.4 (3.4-3.4)
**5-Methyl-tetrahydrofolate**	4.2 (3.5-5.8)	7.4 (5.3-8.5)*	-	-
**5-Formyl-tetrahydrofolate‡**	32.8 (24.8-37.7)	65.6 (39-66.7)*	-	-
**Folic acid**	0.3 (0.3-0.3)	0.1 (0.1-0.1)*	-	-
**Cyanocobalamin‡**	3.4 (2.9-3.8)	2.9 (2.5-3.3)	-	-
**Riboflavin**	10.6 (9.5-13.9)	6.5 (5.7-6.5724)*	0.5 (0.2-0.9)	0.9 (0.9-1)*

Supplementary Table S3. Antibodies used in western blot and immunohistochemical analyses

Antigen	Antibody	1ry Dilution	2ry Antibody	2ry Dilution
**CD11b**	CD11b antibody, ab133357 (Abcam, Cambridge, UK)	1:1000	Goat α-rabbit HRP, P0448 (Dako, Santa Clara, CA, USA)	1:5000
**TNFα**	TNFα antibody, #3707 (Cell signalling, Danvers, MA, USA)	1:1000	Goat α-rabbit HRP, P0448 (Dako, Santa Clara, CA, USA)	1:5000
**CD163**	CD163, antibody, ab182422 (Abcam, Cambridge, UK)	1:1000	Goat α-rabbit HRP, P0448 (Dako, Santa Clara, CA, USA)	1:5000
**F4/80**	F4/80, antibody, ab100790 (Abcam, Cambridge, UK)	1:100	Goat α-rabbit HRP, P0448 (Dako, Santa Clara, CA, USA)	1:200
**Arginase**	Liver arginase, antibody, ab91279 (Abcam, Cambridge, UK)	1:10000	Goat α-rabbit HRP, P0448 (Dako, Santa Clara, CA, USA)	1:5000
**PI3K-p85**	PI3K antibody, #4257 (Cell signalling, Danvers, MA, USA)	1:1000	Goat α-rabbit HRP, P0448 (Dako, Santa Clara, CA, USA)	1:5000
**Akt pS473**	p-Akt antibody, #4060 (Cell signalling, Danvers, MA, USA)	1:1000	Goat α-rabbit HRP, P0448 (Dako, Santa Clara, CA, USA)	1:5000
**Akt**	AKT antibody, #4685 (Cell signalling, Danvers, MA, USA)	1:1000	Goat α-rabbit HRP, P0448 (Dako, Santa Clara, CA, USA)	1:5000
**S6 ps235/236**	pS6 antibody, #4856 (Cell signalling, Danvers, MA, USA)	1:1000	Goat α-rabbit HRP, P0448 (Dako, Santa Clara, CA, USA)	1:5000
**S6**	S6 antibody, #2708 (Cell signalling, Danvers, MA, USA)	1:1000	Goat α-rabbit HRP, P0448 (Dako, Santa Clara, CA, USA)	1:5000
**AMPK**	AMPKα antibody #2532 (Cell signalling, Danvers, MA, USA)	1:1000	Goat α-rabbit HRP, P0448 (Dako, Santa Clara, CA, USA)	1.5000
**AMPK pT172**	pAMPK antibody, #2531 (Cell signalling, Danvers, MA, USA)	1:1000	Goat α-rabbit HRP, P0448 (Dako, Santa Clara, CA, USA)	1:5000
**AMPK**	AMPK antibody #2532S (Cell signalling, Danvers, MA, USA)	1:1000	Goat α-rabbit HRP, P0448 (Dako, Santa Clara, CA, USA)	1:5000
**OXPHOS**	OXPHOS Rodent , antibody, ab ab110413 (Abcam, Cambridge, UK)	1:250	Goat α-mouse HRP, P0447 (Dako, Santa Clara, CA, USA)	1:5000
**LC3**	LC3B Antibody, #2775S (Cell signalling, Danvers, MA, USA)	1:1000	Goat α-rabbit HRP, P0448 (Dako, Santa Clara, CA, USA)	1:5000
**LAMP2A**	LAMP2A antibody, ab125068 (Abcam, Cambridge, UK)	1:1000	Goat α-rabbit HRP, P0448 (Dako, Santa Clara, CA, USA)	1:2000
**P62**	P62 Antibody, #5114 (Cell signalling, Danvers, MA, USA)	1:1000	Goat α-rabbit HRP, P0448 (Dako, Santa Clara, CA, USA)	1:5000
**FAH**	FAH antibody #ABN526 (Millipore, Burlington, MA, USA)	1:1000	Goat α-rabbit HRP, P0448 (Dako, Santa Clara, CA, USA)	1:2000
**Vinculin**	Vinculin antibody, ab73412 (Abcam, Cambridge, UK)	1:1000	Goat α-rabbit HRP, P0448 (Dako, Santa Clara, CA, USA)	1:2000
**PINK1**	PINK1 antibody, ab2370 7(Abcam, Cambridge, UK)	1:1000	Goat α-rabbit HRP, P0448 (Dako, Santa Clara, CA, USA)	1:5000
**PARKIN**	Parkin antibody, #2132 (Cell signalling, Danvers, MA, USA)	1:1000	Goat α-rabbit HRP, P0448 (Dako, Santa Clara, CA, USA)	1:5000
**TOM20**	TOM20 antibody, #42406 (Cell signalling, Danvers, MA, USA)	1:1000	Goat α-rabbit HRP, P0448 (Dako, Santa Clara, CA, USA)	1:5000
**Mfn2**	Mfn2 antibody, ab124773 (Abcam, Cambridge, UK)	1:1000	Goat α-rabbit HRP, P0448 (Dako, Santa Clara, CA, USA)	1:2000

Supplementary Figure S3. Uncropped Western Blots in liver and muscle. The outlined areas correspond to the cropped images shown in the main figures. The aspect ratios have not been altered and the cropped images shown in the main figures and their corresponding originals are versions taken at the same exposure level.

Uncropped blots from Figure 3 liver

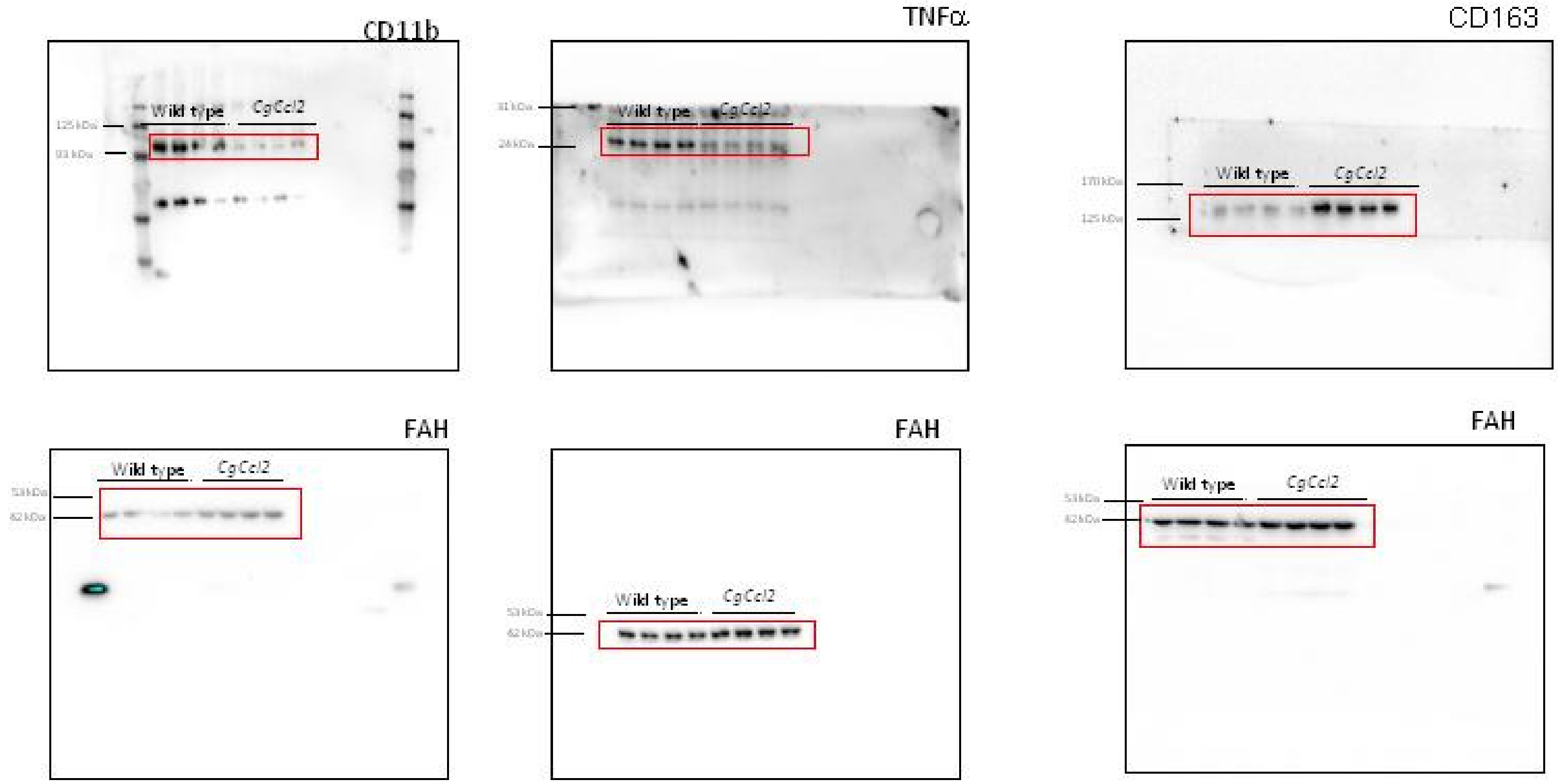

Uncropped blots from Figure 3 muscle

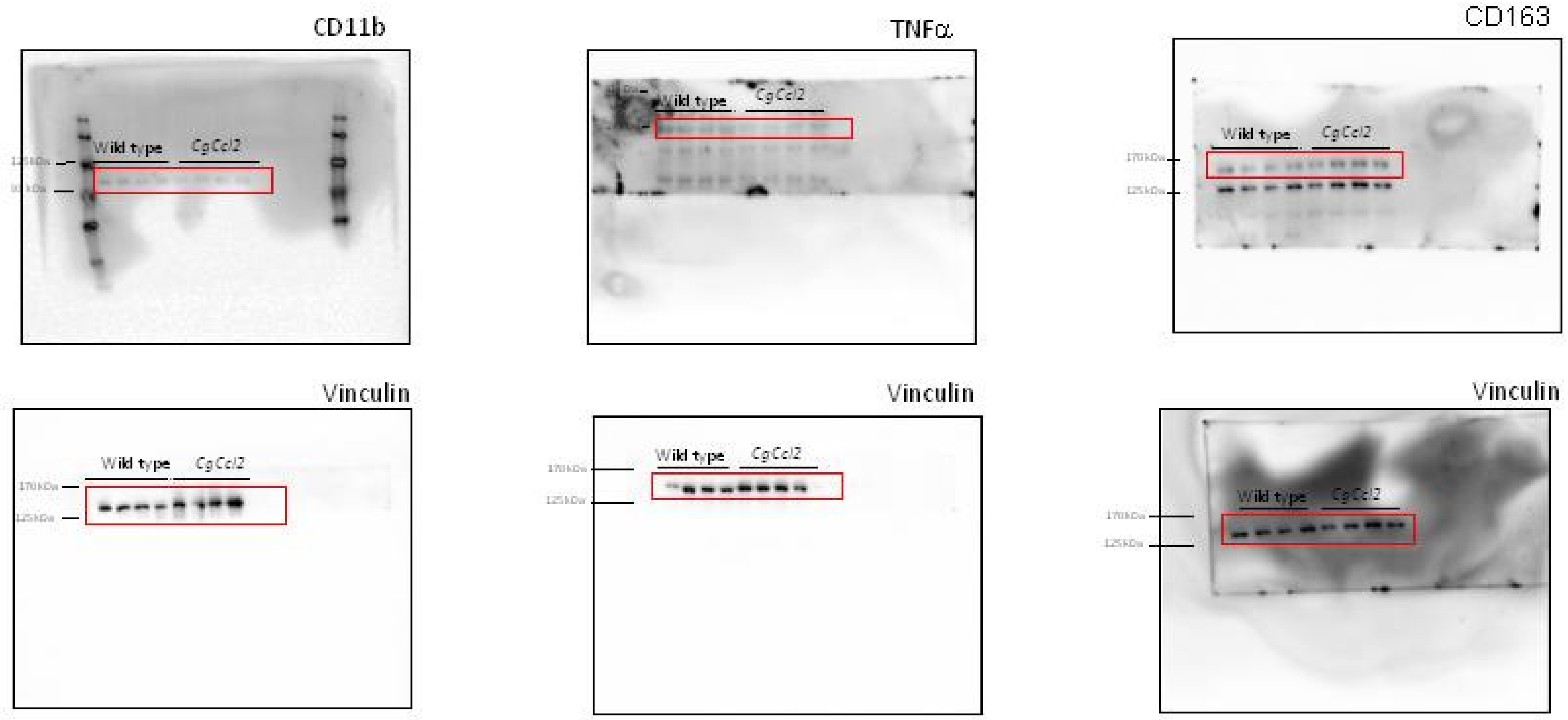

Uncropped blots from Figure 4 Liver

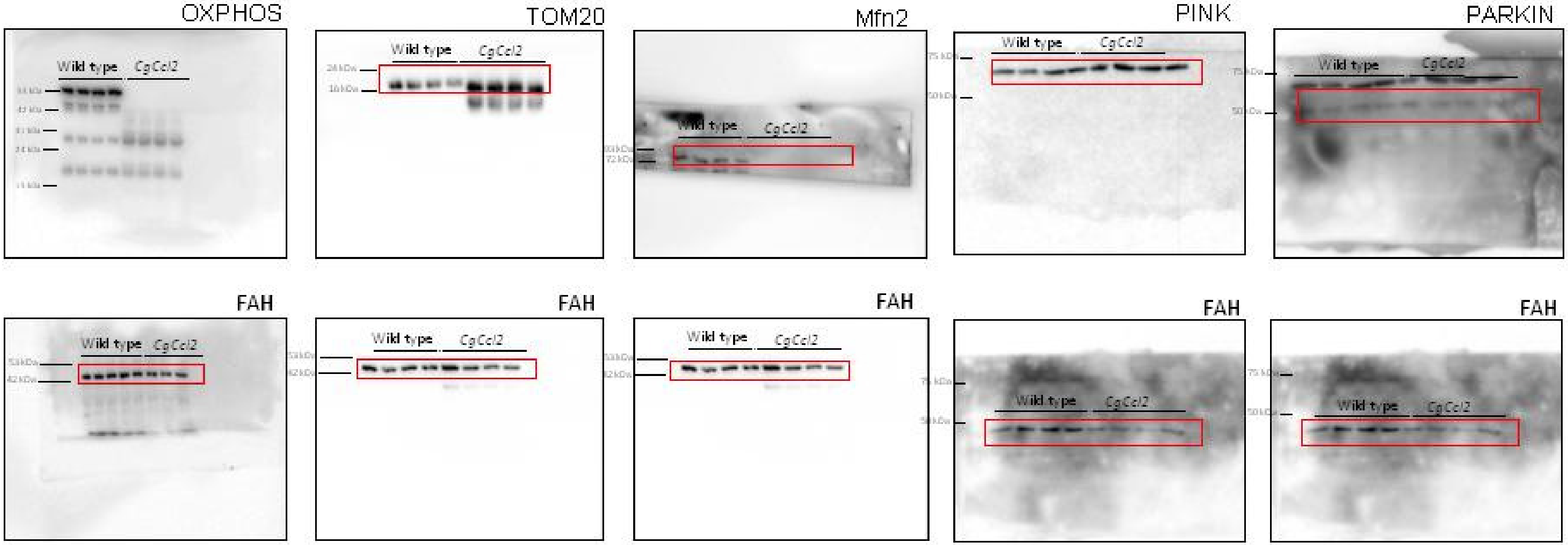

Uncropped blots from Figure 4 Muscle

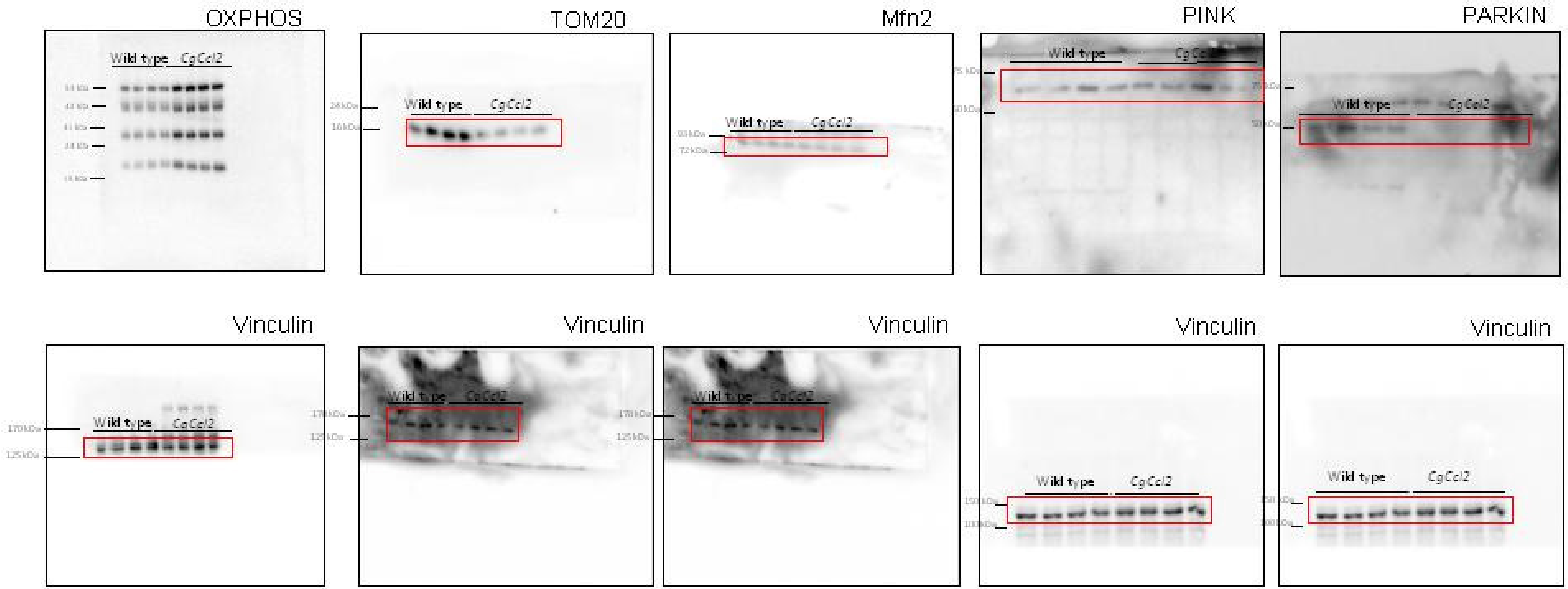

Uncropped blots from Figure 7 Liver

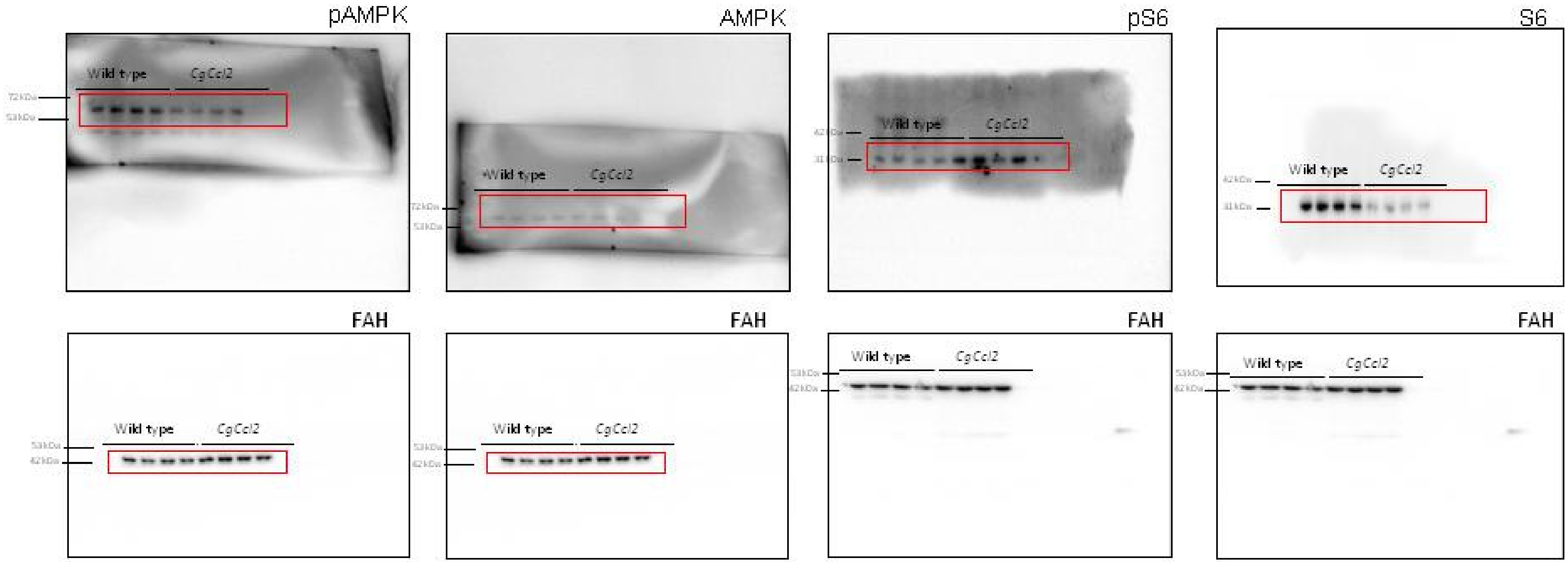

Uncropped blots from Figure 7 Liver

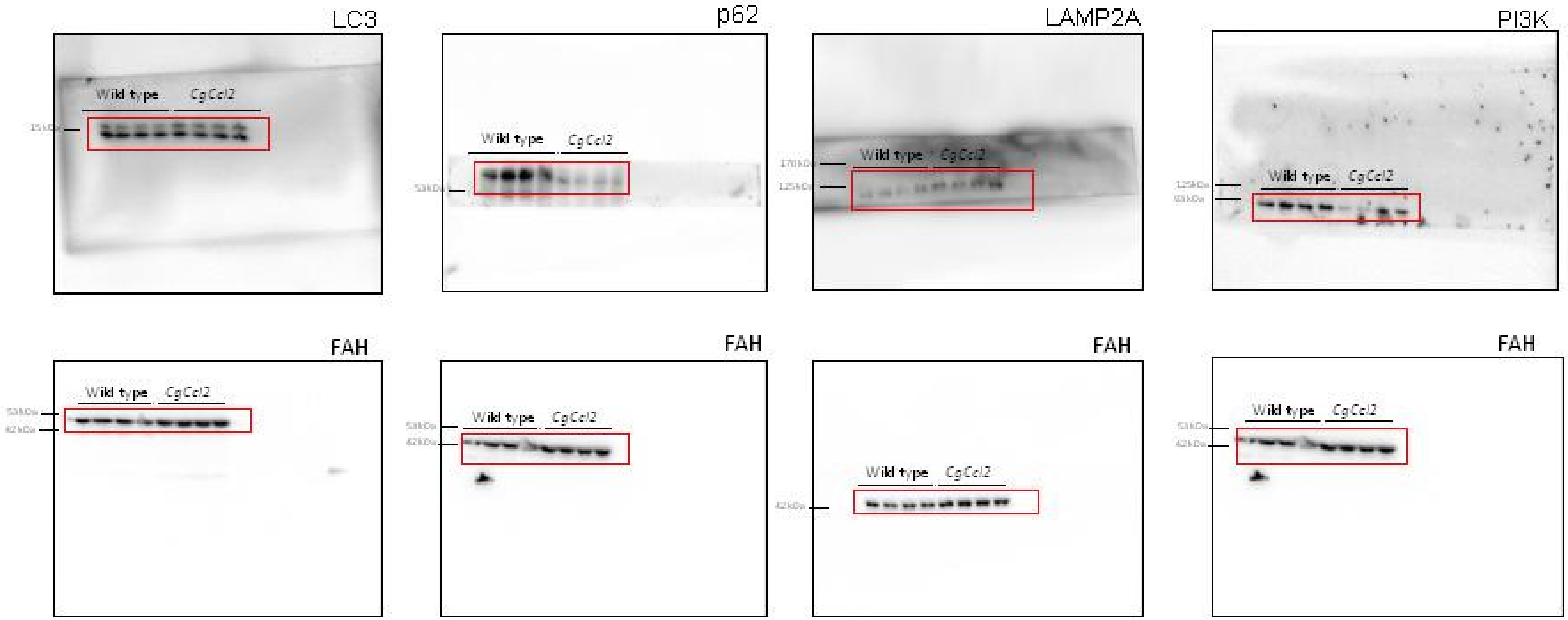

Uncropped blots from Figure 7 Liver

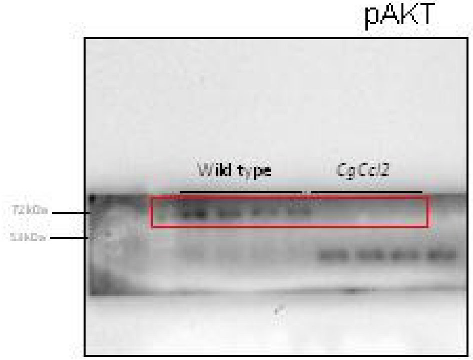

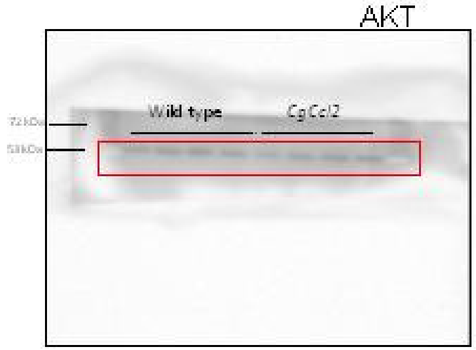

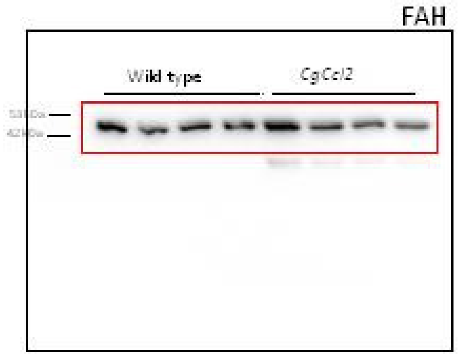

Uncropped blots from Figure 7 Muscle

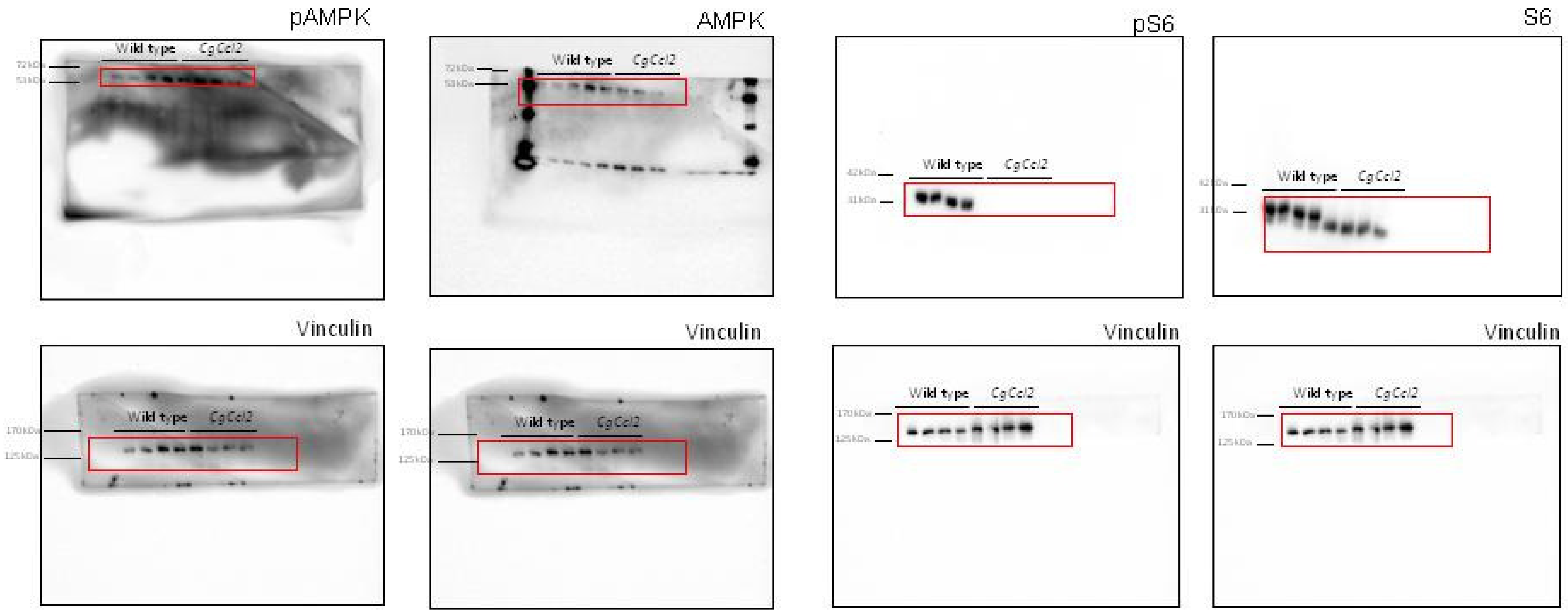

Uncropped blots from Figure 7 Muscle

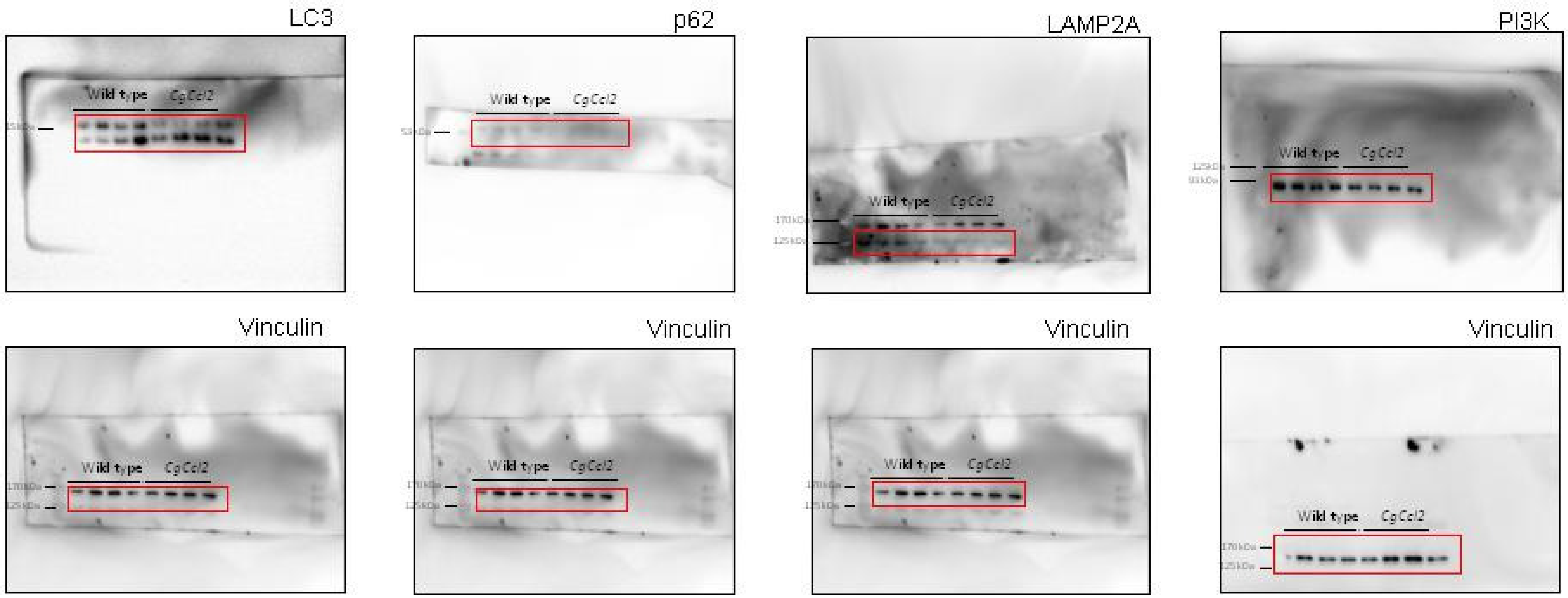

Uncropped blots from Figure 7 Muscle

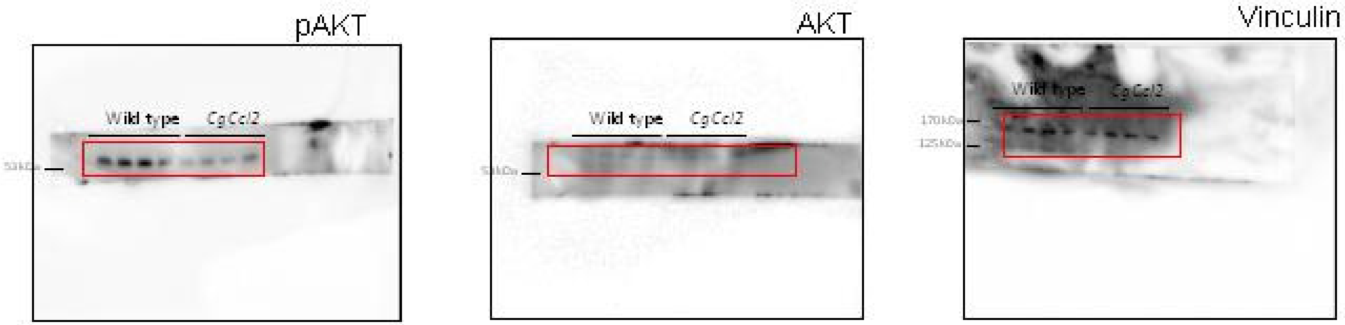

Supplement: Supplementary file 1 — Supplementary file1 (PDF 2148 kb) [file 41598_2020_68769_MOESM1_ESM.pdf]
